# There is more than just longitudinal strain: Prognostic significance of biventricular circumferential mechanics

**DOI:** 10.3389/fcvm.2023.1082725

**Published:** 2023-02-16

**Authors:** Máté Tolvaj, Alexandra Fábián, Márton Tokodi, Bálint Lakatos, Alexandra Assabiny, Zsuzsanna Ladányi, Kai Shiida, Andrea Ferencz, Walter Schwertner, Boglárka Veres, Annamária Kosztin, Ádám Szijártó, Balázs Sax, Béla Merkely, Attila Kovács

**Affiliations:** Semmelweis University Heart and Vascular Center, Budapest, Hungary

**Keywords:** speckle tracking echocardiography, 3D echocardiography, global longitudinal strain, global circumferential strain, heart failure

## Abstract

**Introduction:**

Despite the significant contribution of circumferential shortening to the global ventricular function, data are scarce concerning its prognostic value on long-term mortality. Accordingly, our study aimed to assess both left (LV) and right ventricular (RV) global longitudinal (GLS) and global circumferential strain (GCS) using three-dimensional echocardiography (3DE) to determine their prognostic importance.

**Methods:**

Three hundred fifty-seven patients with a wide variety of left-sided cardiac diseases were retrospectively identified (64 ± 15 years, 70% males) who underwent clinically indicated 3DE. LV and RV GLS, and GCS were quantified. To determine the prognostic power of the different patterns of biventricular mechanics, we divided the patient population into four groups. Group 1 consisted of patients with both LV GLS and RV GCS above the respective median values; Group 2 was defined as patients with LV GLS below the median while RV GCS above the median, whereas in Group 3, patients had LV GLS values above the median, while RV GCS was below median. Group 4 was defined as patients with both LV GLS and RV GCS below the median. Patients were followed up for a median of 41 months. The primary endpoint was all-cause mortality.

**Results:**

Fifty-five patients (15%) met the primary endpoint. Impaired values of both LV GCS (HR, 1.056 [95% CI, 1.027–1.085], *p* < 0.001) and RV GCS (1.115 [1.068–1.164], *p* < 0.001) were associated with increased risk of death by univariable Cox regression. Patients with both LV GLS and RV GCS below the median (Group 4) had a more than 5-fold increased risk of death compared with those in Group 1 (5.089 [2.399–10.793], *p* < 0.001) and more than 3.5-fold compared with those in Group 2 (3.565 [1.256–10.122], *p* = 0.017). Interestingly, there was no significant difference in mortality between Group 3 (with LV GLS above the median) and Group 4, but being categorized into Group 3 versus Group 1 still held a more than 3-fold risk (3.099 [1.284–7.484], *p* = 0.012).

**Discussion:**

The impaired values of both LV and RV GCS are associated with long-term all-cause mortality, emphasizing the importance of assessing biventricular circumferential mechanics. Reduced RV GCS is associated with significantly increased risk of mortality even if LV GLS is preserved.

## Introduction

Longitudinal strain (LS) of the left (LV) and right ventricles (RV) is a well-established biomarker of ventricular dysfunction, having a robust predictive power to future adverse outcomes in numerous cardiac diseases (1, 2). In contrast to ejection fraction (EF), a global measure of pump function, LS enables the quantification of ventricular deformation along their long axis. As many cardiac disease processes primarily affect the mainly longitudinally-oriented subendocardial layer, the measurement of LS is more sensitive to subtle changes and possesses added prognostic power compared to EF (2, 3).

Besides longitudinal shortening, also circumferential deformation contributes significantly to the ventricular pump function (4, 5). Thus, global ventricular function is the resultant of these two distinct but interconnected deformation components. Despite the unequivocal importance of circumferential shortening in biventricular function, data are scarce concerning its prognostic value on long-term mortality.

The advantages of three-dimensional echocardiography (3DE) over conventional echocardiography opens the door for a more thorough understanding and quantification of the ventricular structure and function, including the measurement of the circumferential strain of both the LV and the RV.

Although the detailed assessment of RV function is commonly neglected in left-sided heart diseases, it has been proved that patients who develop RV dysfunction are more symptomatic and carry a higher risk for long-term adverse outcomes than those who do not (6). Importantly, the prognostic value of RV function was also found to be independent of LV function in these patients (7). Therefore, the detailed assessment of the RV mechanics has increasing importance in identifying patients at risk for developing right heart failure and subsequent adverse outcomes.

Accordingly, we aimed to assess LV and RV GLS and GCS using 3DE to determine their prognostic relevance.

## Methods

### Study design and population

Clinically and hemodynamically stable patients with an established diagnosis of left-sided cardiac disease were identified from the previously published RVENet dataset [https://rvenet.github.io/dataset/], which comprises of individuals underwent clinically indicated 2D and 3D transthoracic echocardiography at our Center between November 2013 and March 2021. Exclusion criteria were 1) suspicion or presence of any primary right-sided cardiac disease at the first report or during the review process of the previously acquired datasets and 2) suboptimal LV and RV 3D dataset image quality for the respective 3D analysis. Demographic and clinical data (age, body surface area, body mass index, systolic and diastolic blood pressure, heart rate, cardiovascular risk factors, comorbidities, medical history, and laboratory parameters) were retrieved from the electronic clinical records. Obtaining written informed consent was waived due to the retrospective nature of the analysis. Our study protocol follows the Declaration of Helsinki and it was approved by the Semmelweis University Regional and Institutional Committee of Science and Research Ethics (approval No. 190/2020).

### Two- and three-dimensional echocardiography

Transthoracic echocardiographic examinations were performed on commercially available ultrasound systems (E95, 4Vc-D probe, GE Vingmed Ultrasound, Horten, Norway, and EPIQ 7, X5-1 probe, Philips Medical Systems, Best, the Netherlands). A standard acquisition protocol consisting of 2D loops from parasternal, apical, and subxiphoid views was applied. LV internal diameters, wall thicknesses, relative wall thickness, and mass; left atrial (LA) 2D end-systolic volume; mitral inflow velocities such as early (E) and late diastolic (A) peak velocities, their ratio, and E wave deceleration time; systolic (s′), early diastolic (e′), and atrial (a′) velocities of the mitral lateral and septal annulus; average E/e′; RV basal short-axis diameter, tricuspid annular plane systolic excursion (TAPSE), fractional area change (FAC); right ventricular systolic pressure (RVSP) and right atrial (RA) 2D end-systolic volume were measured according to current guidelines (8).

Beyond conventional echocardiographic examination, ECG-gated full-volume 3D datasets reconstructed from four cardiac cycles optimized for the left or the right heart were obtained for further analysis on a separate workstation. 3D datasets focused on the left heart were processed using semiautomated, commercially available software (4D LV-Analysis 3, TomTec Imaging, Unterschleissheim, Germany). We determined LV end-diastolic volume index (EDVi), end-systolic volume index (ESVi), stroke volume index (SVi), and mass index (Mi). To assess global LV function, ejection fraction (EF), 3D global longitudinal strain (GLS), and 3D global circumferential strain (GCS) were also calculated. Concerning the right heart, we quantified 3D RV EDVi, ESVi, SVi, EF, and septal and free wall two-dimensional longitudinal strain as well (4D RV-Function 2, TomTec Imaging). Using the ReVISION software (Argus Cognitive, Inc., Lebanon, NH, USA), we have quantified 3D RV GLS and GCS as previously described (9). By convention, GLS and GCS values are negative, meaning that less negative values refer to more impaired ventricular function.

### Study outcomes

The patients were followed up for a maximum of 6 years. Follow-up data (status [dead or alive], date of death) was obtained from Hungary's National Health Insurance Database. The primary endpoint of our study was all-cause mortality.

### Statistical analysis

Statistical analysis was performed using SPSS (v22, IBM, Armonk, NY, USA) and R (version 3.6.2, R Foundation for Statistical Computing, Vienna, Austria). Continuous variables are expressed as mean ± standard deviation (SD), whereas categorical variables were reported as frequencies and percentages. After verifying the normal distribution of variables using the Shapiro-Wilk test, the clinical and echocardiographic characteristics were compared with unpaired Student's *t*-test or Mann-Whitney *U*-test for continuous variables, and Chi-squared or Fisher's exact test for categorical variables, as appropriate. Multiple group comparisons (>2) were performed using ANOVA (with Tukey *post-hoc* test) or Kruskal-Wallis test (with Dunn *post-hoc* test) and χ2 or Fisher exact test, as appropriate. Using univariable Cox regression, we identified factors associated with all-cause mortality. Targeting a maximum of 1 covariate per 10 events, we built several sequential multivariable Cox proportional hazards models. First, we constructed a baseline model including only clinical and laboratory parameters, and then in two consecutive steps, we added different LV and RV functional parameters to the model. As the final step, the constructed multivariable models were compared based on Akaike Information Criterion (AIC) to determine which one is the best fit for our data. Collinearity was tested using the variance inflation factor (excessive if variance inflation factor >3). Survival of the subgroups was visualized *via* Kaplan-Meier curves and compared using log-rank tests. Cox proportional hazards models were used to compute hazard ratios (HRs) with 95% confidence intervals (95% CIs) between the groups. Receiver-operating characteristic (ROC) curves were constructed to investigate the discriminative power of 2D and 3D echocardiographic parameters with regards to the primary endpoint. Metrics having more than 10% of missing values were not included in these analyses. Intraobserver and interobserver variability were also tested: the first reader repeated the analysis in a randomly chosen subset of patients (*n* = 15) blinded to previous results. A second reader also analyzed this patient subset in a blinded fashion. Intraclass correlation coefficient values were calculated. A two-sided *P*-value of < 0.05 was considered statistically significant.

## Results

### Baseline clinical characteristics

Three hundred fifty-seven patients (age: 64 ± 15 years, 70% males) with established left-sided cardiac disease and 3DE recordings suitable for LV and RV analysis were identified from the RVENet dataset (444 patients were initially identified of whom we excluded 80 due to inadequate 3D image quality for RV analysis, and further 7 due to inadequate image quality for LV analysis). During the median follow-up time of 41 months (interquartile range 20–52), 55 (15%) patients died. Demographics and clinical characteristics of the study cohort and a comparison of patients alive vs. those who died are presented in Table 1.

**Table 1:** Demographic and clinical characteristics.

	**Overall (*n* = 357)**	**Alive (*n* = 302)**	**Dead (*n* = 55)**	***p***
**Baseline demographic characteristics**				
Age (years)	64.2 ± 14.5	63.4 ± 14.6	68.6 ± 13.1	**0.014**
Male, *n* (%)	249 (69.7)	211 (69.9)	38 (69.1)	0.908
BSA (m^2^)	1.93 ± 0.22	1.93 ± 0.22	1.91 ± 0.22	0.494
BMI (kg/m^2^)	26.8 ± 4.2	27.0 ± 4.3	26.0 ± 3.6	0.288
Systolic blood pressure (mmHg)	126.5 ± 18.4	126.1 ± 17.2	128.0 ± 23.2	0.585
Diastolic blood pressure (mmHg)	74.6 ± 15.3	74.0 ± 15.9	77.4 ± 12.3	0.230
Heart rate (bpm)	77.6 ± 14.6	77.7 ± 15.0	77.4 ± 12.5	0.935
**Risk factors and medical history**				
Hypertension, *n* (%)	260 (72.8)	218 (72.2)	42 (76.4)	0.522
History of smoking, *n* (%)	82 (23.0)	66 (21.9)	16 (29.1)	0.241
COPD, *n* (%)	40 (11.2)	33 (10.9)	7 (12.7)	0.735
Diabetes, *n* (%)	99 (27.7)	78 (25.8)	21 (38.2)	0.060
History of atrial fibrillation, *n* (%)	116 (32.5)	89 (29.5)	27 (49.1)	**0.005**
PM, *n* (%)	49 (13.7)	38 (12.6)	11 (20.0)	0.159
ICD, *n* (%)	33 (9.2)	23 (7.6)	10 (18.2)	**0.015**
CRT, *n* (%)	15 (4.2)	12 (4.0)	3 (5.5)	0.637
CAD, *n* (%)	77 (21.6)	55 (18.2)	22 (40.0)	**< 0.001**
Previous CABG, *n* (%)	19 (5.3)	13 (4.3)	6 (10.9)	**0.045**
Previous PCI, *n* (%)	67 (18.8)	49 (16.2)	18 (32.7)	**0.004**
Previous AMI, *n* (%)	48 (13.4)	34 (11.3)	14 (25.5)	**0.005**
**Laboratory parameters**				
GFR (mL/min/1.73m^2^)	61.0 ± 19.4	62.1 ± 19.1	56.3 ± 20.2	0.056
Creatinine (μmol/L)	101.1 ± 41.7	99.1 ± 38.6	112.1 ± 54.5	**0.035**
Hgb (g/dL)	12.9 ± 2.1	12.9 ± 2.1	12.6 ± 2.2	0.385
CRP (mg/L)	6.7 ± 11.9	6.3 ± 12.1	9.0 ± 10.5	0.134

Continuous variables are presented as means ± SD, categorical variables are reported as frequencies (%). AMI, acute myocardial infarction; BMI, body mass index; BSA, body surface area; CABG, coronary artery bypass grafting; CAD, coronary artery disease; COPD, chronic obstructive pulmonary disease; CRP, C-reactive protein; CRT, cardiac resynchronization therapy; GFR, glomerular filtration rate; Hgb, hemoglobin; ICD, implantable cardioverter defibrillator; PCI, percutaneous coronary intervention; PM, pacemaker. Bold values mean significant *p* values (below 0.05).

Ninety-five subjects (27%) were heart failure with reduced ejection fraction (HFrEF) patients, of whom 81 patients were referred to our electrophysiology department for assessment prior to device implantation (pacemaker/implantable cardioverter defibrillator [ICD]/cardiac resynchronization therapy [CRT]). Fourteen patients were investigated for candidacy for a long-term LV assist device (LVAD) implantation. Ninety-one subjects (26%) were heart transplant recipients (HTX) with a median of 157 days after the operation (ranging from 8 to 6,571 days). Sixty-seven subjects (19%) were patients with severe primary mitral valve regurgitation (MVR) enrolled in a previous prospective study (10). Seventy-nine patients (22%) were investigated to evaluate aortic stenosis severity (moderate or severe). Twenty-five patients (7%) with a history of atrial fibrillation were referred for evaluation before a potential catheter ablation. The most frequently observed comorbidities were hypertension (73%), diabetes (28%), coronary artery disease (22%), and atrial fibrillation (33%).

Patients who died were older, had a higher prevalence of coronary artery disease and atrial fibrillation, and more frequently underwent ICD implantation in their medical history. Importantly, these patients had higher serum creatinine values than those who survived (Table 1).

### Echocardiographic characteristics

2D echocardiographic parameters are summarized in Supplementary Table 1. Interestingly, conventional morphological parameters of the LV, the LA, and the RV did not differentiate between patients who died vs. those who survived. Mitral annular velocities by TDI, both in systole and diastole, were more impaired in those patients who died. However, E/e' was similar. The right atrial size was larger in those patients who died, along with a more impaired RV longitudinal function (TAPSE, free wall longitudinal strain); however, RV systolic pressure and FAC were similar.

3DE parameters are summarized in Table 2. Patients who died had larger LV and RV volumes, along with a more impaired systolic function. Notably, LV SVi and RV SVi were similar. In terms of longitudinal and circumferential biventricular strains, both LV and RV GLS and GCS were more impaired in patients who died.

**Table 2:** 3D echocardiographic parameters.

	**Overall (*n* = 357)**	**Alive (*n* = 302)**	**Dead (*n* = 55)**	***p***
**Left ventricle**				
LV EDVi (ml/m^2^)	82.2 ± 32.2	80.3 ± 32.3	91.9 ± 30.3	**0.019**
LV ESVi (ml/m^2^)	44.5 ± 30.4	42.2 ± 30.0	56.3 ± 30.0	**0.003**
LV SVi (ml/m^2^)	37.7 ± 14.6	38.1 ± 15.1	35.6 ± 11.2	0.269
LV Mi (g/m^2^)	102.5 ± 36.8	100.4 ± 35.1	113.4 ± 43.0	**0.023**
LV EF (%)	49.0 ± 15.7	50.2 ± 15.3	42.3 ± 16.1	**0.001**
LV GLS (%)	−15.2 ± 6.0	−15.7 ± 5.9	−12.5 ± 6.2	**< 0.001**
LV GCS (%)	−23.9 ± 9.1	−24.6 ± 9.0	−20.2 ± 9.3	**0.001**
**Right ventricle**				
RV EDVi (ml/m^2^)	70.2 ± 23.5	68.9 ± 23.1	76.6 ± 24.6	**0.033**
RV ESVi (ml/m^2^)	37.4 ± 18.7	36.0 ± 17.8	44.4 ± 21.3	**0.003**
RV SVi (ml/m^2^)	32.7 ± 9.0	32.8 ± 9.3	32.2 ± 7.5	0.648
RV EF (%)	48.3 ± 9.4	49.1 ± 9.2	44.1 ± 9.5	**< 0.001**
RV GLS (%)	−16.4 ± 5.1	−16.9 ± 5.0	−13.8 ± 4.6	**< 0.001**
RV GCS (%)	−17.7 ± 6.1	−18.3 ± 5.9	−14.3 ± 6.2	**< 0.001**

Continuous variables are presented as means ± SD, categorical variables are reported as frequencies (%). EDVi, end-diastolic volume index; EF, ejection fraction; ESVi, end-systolic volume index; GCS, global circumferential strain; GLS, global longitudinal strain; LV, left ventricle; Mi, mass index; RV, right ventricle; SVi, stroke volume index. Bold values mean significant *p* values (below 0.05).

**Figure 1:**
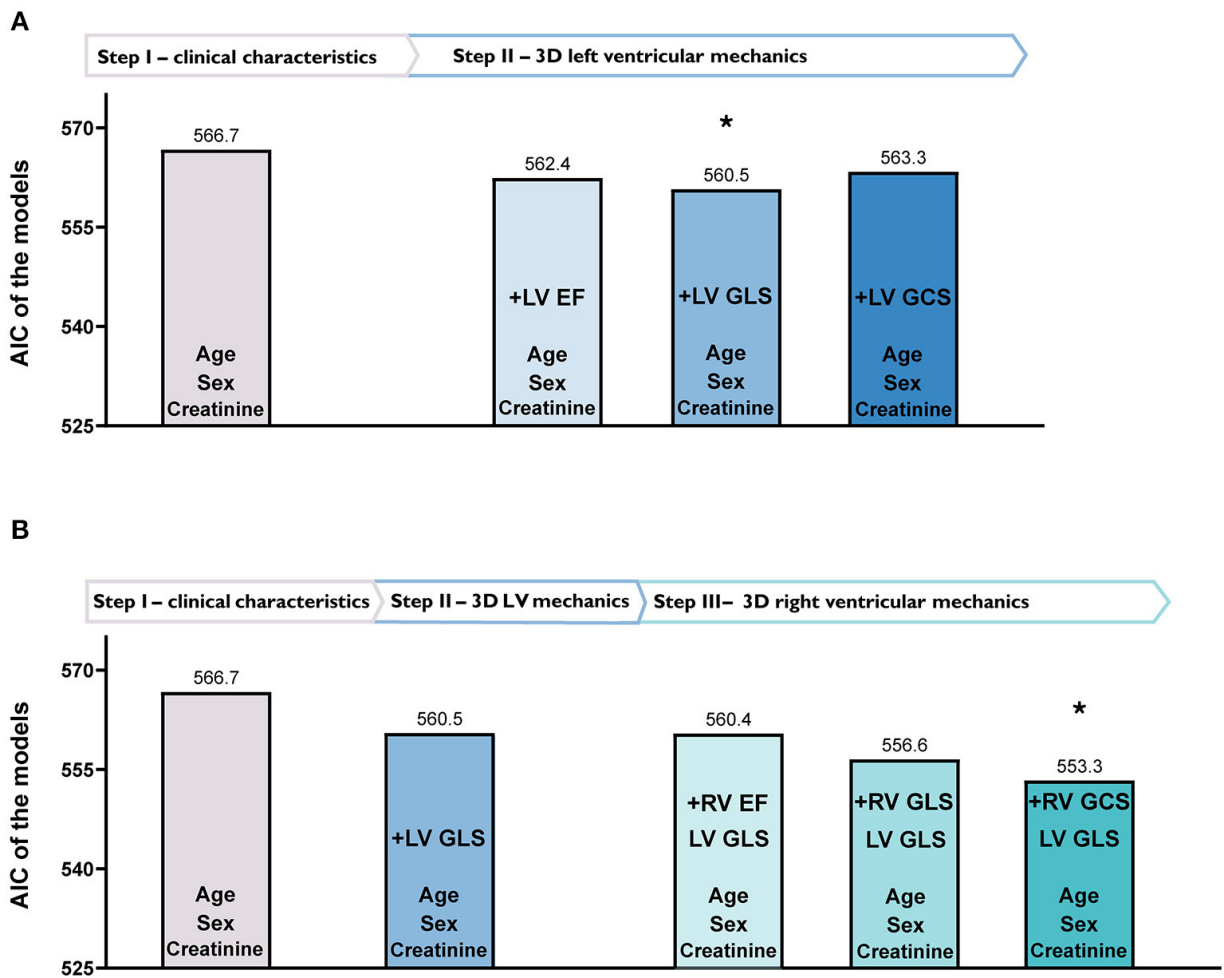
Identification of the best-fit models, including left (LV) and right ventricular (RV) functional parameters by multivariable Cox regression analysis based on Akaike Information Criterion (AIC). **(A)** Depicts different models with only clinical characteristics (Step I) and 3D LV mechanical parameters added one by one (Step II). In Step II, adding LV global longitudinal strain (GLS) to the model resulted in the lowest (best) AIC value. **(B)** Shows the added value of 3D RV mechanical parameters (Step III). In Step III, adding RV global circumferential strain (GCS) to the previously established model in Step II (clinical characteristics and LV GLS) resulted in the best fit to our data as confirmed by the lowest AIC value.

Using univariable Cox regression, we identified variables associated with all-cause mortality (Supplementary Table 2). Then, we created several multivariable Cox models with a maximum of 5 predictors by adding covariates to a baseline model in a sequential manner (Figure 1). This analysis comprised three consecutive steps. In the first step, we created a baseline model (Model 0) that included age, sex, and serum creatinine level, as that latter was found to be a significant predictor during univariable analysis. In the second step, we added LVEF, LV GLS, or LV GCS to the baseline model one by one (Model 1, Model 2, and Model 3, respectively), and we found that the model with LV GLS (Model 2) had the lowest AIC among the constructed models (Figure 1A). In the third step, we built Models 4, 5, and 6 by adding RV EF, RV GLS, or RV GCS to Model 2, respectively. Among these models, the one with RV GCS (Model 6) exhibited the lowest AIC (Figure 1B and Supplementary Table 3). In this model, age and RV GCS were independent predictors of all-cause mortality, whereas sex, creatinine level, and LV GLS were not (Table 3). We have confirmed that our approach identified the best combination of covariates by constructing multivariable models with all possible combination of the LV and RV parameters (Supplementary Table 4). In multivariable models including the medical history of coronary artery disease or atrial fibrillation instead of sex, RV GCS remained an independent predictor of all-cause mortality (Supplementary Tables 5, 6). On ROC analysis, LV GLS had the highest discriminative power among LV functional parameters (area under the ROC curve: 0.644 [95% CI: 0.561–0.726, *p* < 0.001]), nevertheless, RV GCS had the highest discriminative power among all the investigated 2D and 3D echocardiographic parameters (0.690 [95% CI: 0.614–0.765, *p* < 0.001], Supplementary Table 7).

**Table 3:** Independent predictors of all-cause mortality identified using multivariable cox regression.

**Multivariable cox regression**		
	**HR [95% CI]**	***p***
Age	1.036 [1.011–1.061]	**0.004**
Sex	0.690 [0.376–1.266]	0.231
Creatinine^#^	1.005 [0.999–1.012]	0.087
LV GLS	1.017 [0.963–1.075]	0.543
RV GCS	1.091 [1.032–1.152]	**0.002**

^#^Creatinine levels were available in 341 patients.

CI, confidence interval; HR, hazard ratio; LV GLS, left ventricular global longitudinal strain; RV GCS, right ventricular global circumferential strain. Bold values mean significant *p* values (below 0.05).

### Subgroup analysis

As the model, which included LV GLS and RV GCS, was identified as the best among the evaluated models, we created four subgroups based on the median values of LV GLS and RV GCS (−15.9 and −17.9%, respectively). In Group 1, patients had both LV GLS and RV GCS above the median, while in Group 4, both LV GLS and RV GCS were below the median. Group 2 patients had LV GLS values below the median while RV GCS above the median. Group 3 had patients with LV GLS above the median and RV GCS below the median.

Out of the 125 patients in Group 1, 37 (30%) were HTX recipients, 19 (15%) were evaluated before atrial fibrillation ablation, 18 (14%) had aortic stenosis, and 51 (41%) had mitral valve disease. Group 2 consisted of 53 patients, of whom 16 (30%) had HFrEF, 11 (21%) were HTX recipients, 6 (11%) were evaluated before atrial fibrillation ablation, and 20 (38%) had aortic stenosis. Group 3 had 54 patients in total, one (2%) with HFrEF, 30 (56%) HTX recipients, 8 (15%) with aortic stenosis, and 15 (28%) with mitral valve disease. Group 4 was composed of 125 patients and had 78 (62%) HFrEF patients, 13 (10%) HTX recipients, 33 (26%) patients with aortic stenosis, and 1 (1%) with mitral valve disease.

**Figure 2:**
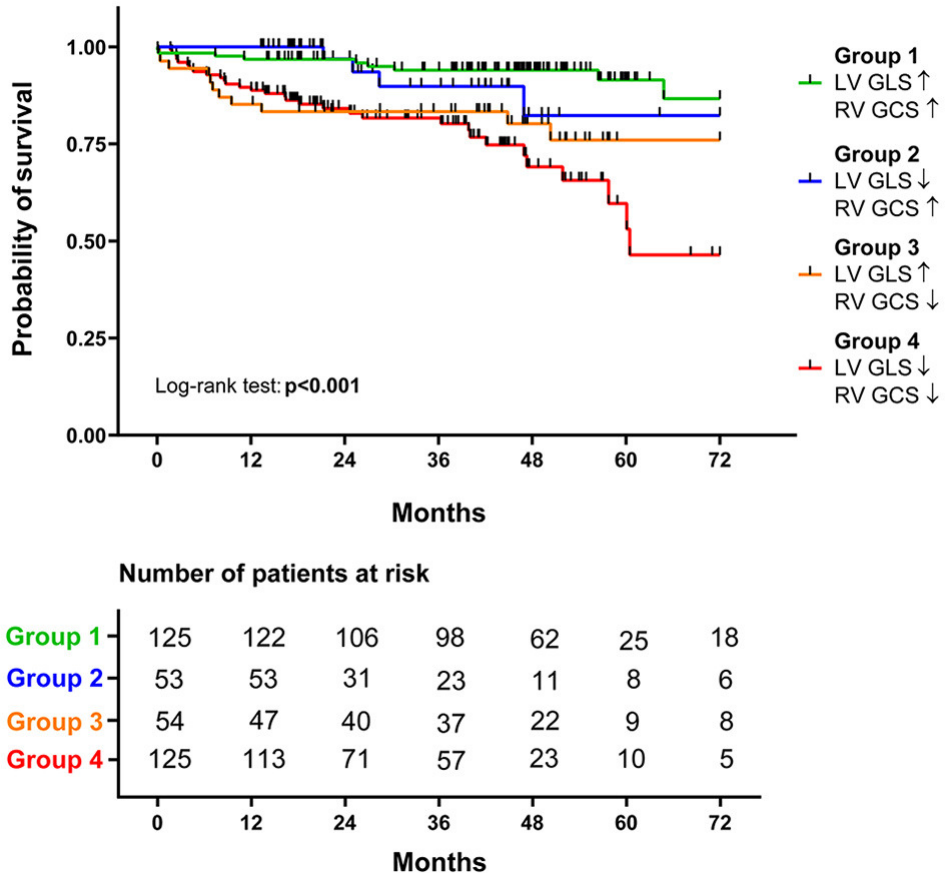
Survival analysis of the different groups. Based on the respective median values of left ventricular global longitudinal strain (LV GLS, −15.9%) and right ventricular global circumferential strain (RV GCS, −17.9%), patients were divided into four groups. The survival of the four groups is visualized on Kaplan-Meier curves, and the log-rank test was performed for comparison.

In Group 1, 7.2% of the patients died during the follow-up, while 7.5% died in Group 2. Group 3 and Group 4 patients experienced adverse outcomes more frequently, with 20.3% mortality in the former and 24.8% in the latter. These differences in the outcomes between patient subgroups were visualized *via* Kaplan-Meier curves (Figure 2). Patients with both LV GLS and RV GCS below the median (Group 4) had a more than 5-fold increased risk of death compared with those in Group 1 and more than 3.5-fold compared with those in Group 2. Interestingly, there was no significant difference in mortality between Group 3 (with LV GLS above the median) and Group 4, but being categorized into Group 3 vs. Group 1 still held a more than 3-fold risk (Supplementary Table 8).

Regarding the baseline characteristics of these groups, Group 4 patients were older, presented with lower systolic blood pressure, more frequently had diabetes and coronary artery disease, and had ICD implantation in their medical history (Supplementary Table 9). Their creatinine levels were also the highest among the groups. On the contrary, in Group 3, there were relatively younger patients, less frequently with diabetes, coronary artery disease, or atrial fibrillation in their medical history. Group 4 patients were presented with the highest LV, RV, and RA dimensions and E/e', while LAVi or RV systolic pressure did not differ between the four subgroups (Supplementary Table 10).

The 3DE parameters of the four groups are demonstrated in Supplementary Table 11. Beyond the aforementioned significant chamber dilation and biventricular functional impairment seen in Group 4 patients, Group 3 patients had the lowest LV Mi with a preserved average LV and RV EF (60 ± 5 and 49 ± 6%, respectively).

### Reproducibility

Intraclass correlation coefficient values were lower but still acceptable for RV GCS compared with RVEF and RV GLS (Supplementary Table 12).

## Discussion

**Figure 3:**
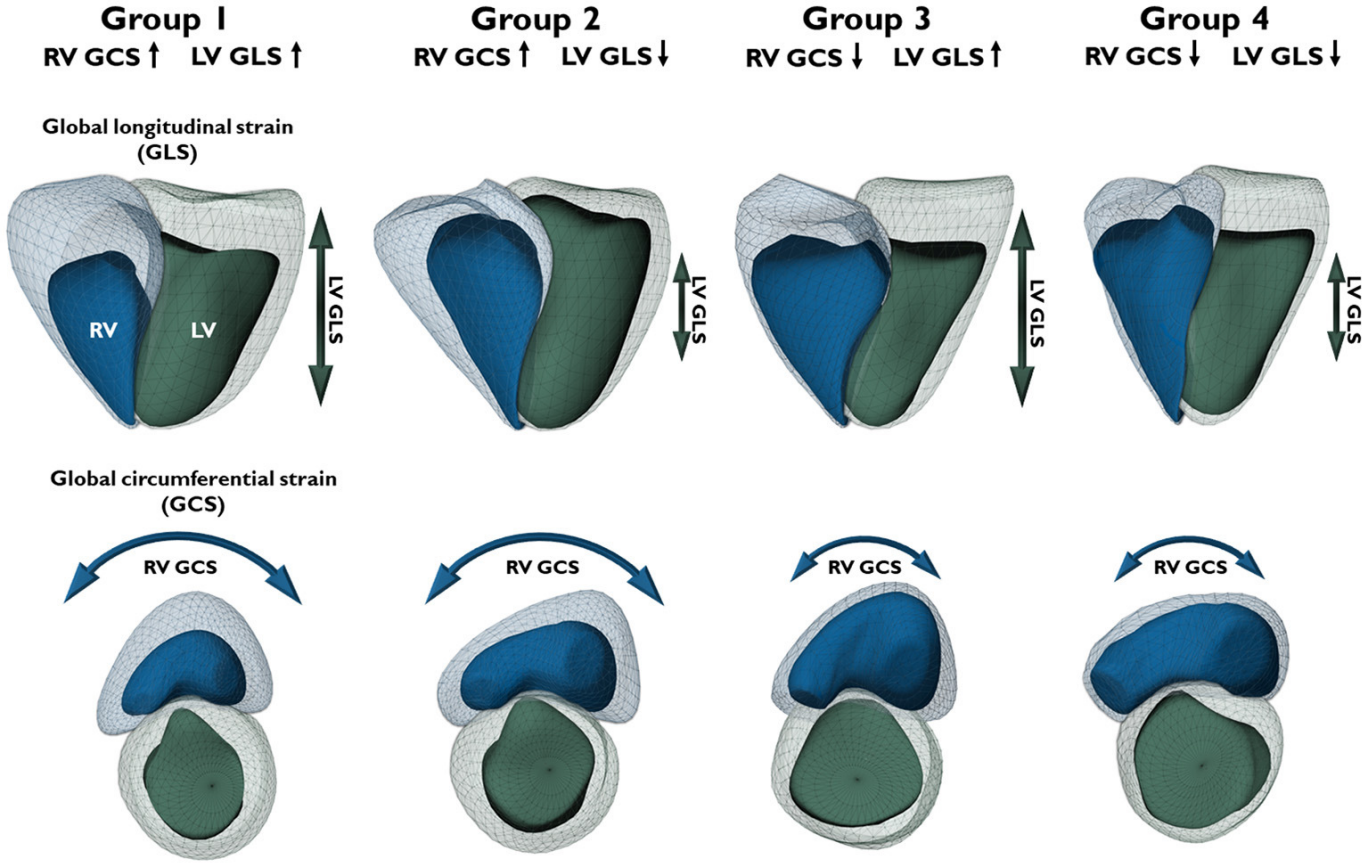
Three-dimensional schematic models depict representative cases of different biventricular mechanical patterns in patients from the respective groups. The four groups were divided based on the median values of left ventricular global longitudinal strain (LV GLS, −15.9%) and right ventricular global circumferential strain (RV GCS, −17.9%). Upward arrows represent strain values better than the median (more negative), downward arrows represent strain values worse than the median (less negative). Light green mesh – left ventricular end-diastolic volume; dark green surface – left ventricular end-systolic volume; green arrow – LV longitudinal shortening (GLS); light blue mesh – right ventricular end-diastolic volume; dark blue surface – right ventricular end-systolic volume; blue arrow – RV circumferential shortening (GCS).

To the best of our knowledge, our study is the first that specifically aimed to assess the prognostic value of both LV and RV circumferential shortening using 3DE. Our main findings can be summarized as follows: (1) Impaired values of both LV and RV GCS were associated with all-cause mortality in univariable Cox models. (2) Among the evaluated multivariable models, the one with LV GLS and RV GCS fitted our data the best. Interestingly, however, only RV GCS was found to be an independent predictor of mortality, whereas LV GLS and creatinine levels were not. (3) Based on the median values of LV GLS and RV GCS, we created four groups that differed significantly in terms of all-cause mortality. Importantly, reduced RV GCS was associated with worse outcomes even if LV GLS was maintained (Figure 3).

Recent advances in echocardiographic hardware and software environment enabled the automated and accurate quantification of myocardial mechanics. First and foremost, GLS by 2D speckle tracking echocardiography has emerged as a mainstay parameter of LV systolic function. Due to the representation of subendocardial longitudinally-oriented myofibers, LV GLS is more sensitive to subtle functional impairment in various clinical scenarios than conventional echocardiographic parameters (i.e., LV EF). A meta-analysis comprising 16 published articles provided strong evidence about the prognostic value of LV GLS, which appeared to be superior to LV EF for predicting major adverse cardiac events in patients with different underlying cardiac abnormalities. GLS is now a well-validated and reproducible metric for the quantification of LV longitudinal deformation, and its integration into routine clinical practice is about to be completed in the upcoming years (2).

Nevertheless, besides longitudinal shortening, circumferential shortening also contributes significantly to the global systolic function of both ventricles. A mathematical model showed that LV GCS contributes more than twice as much to LV EF than LV GLS, and a small increase in LV GCS could compensate for a significant reduction of LV GLS (5). LV EF was shown to be quadratically dependent on circumferential shortening and only linearly dependent on longitudinal shortening. Previously, numerous publications reported global circumferential strain by 2D echocardiography; however, due to its complicated calculation (3 levels of parasternal short-axis view are needed to be acquired and analyzed) and poor reproducibility, the majority of software vendors discontinued the possibility of its measurement (11, 12). Using 3DE, a single acquisition (and the same cardiac cycle) can be used to calculate both GCS and GLS; thus, it may overcome the limitations of 2D-based calculations.

Importantly, circumferential shortening contributes substantially to global RV pump function, which can be measured only by using 3DE. Notably, the circumferential motion may also hold particular importance in the global RV function: even subtle deterioration in the circumferential shortening of the large RV free wall may result in significant global functional damage (13). Circumferential shortening is composed of the inward motion of the RV free wall (radial shortening) and the traction of the free wall insertion lines toward each other by the LV contraction (anteroposterior shortening) (14). Beyond the latter functional connection of the two ventricles, evidence suggests that LV-RV interactions exist on many layers: alterations of geometry, loading conditions, and contractility of a ventricle will significantly influence its counterpart (15). This complex interplay underpins that practically every disease process may have consequences on both sides of the interventricular septum, with potential importance in terms of diagnosis and outcomes.

With the constantly increasing recognition of the left-right heart interaction, a detailed assessment of the right ventricle gains more and more momentum. This approach is hugely facilitated by the advancements of 3DE imaging, allowing real-time examination of the right ventricle, free of geometrical assumptions. 3D RV analysis enables the calculation of RV volumes and, therefore, RV EF. 3D RV EF by itself holds an established added clinical value: as of today, multiple clinical studies have demonstrated the independent prognostic importance of the parameter. Surkova et al. performed their retrospective study on a large cohort of patients with different cardiovascular diseases (7). Their study found that reduced RV EF was independently associated with all-cause mortality and cardiac death after adjusting for clinical and echocardiographic parameters. RV EF also demonstrated higher sensitivity and specificity for predicting all-cause mortality than conventional parameters of RV systolic function (TAPSE and FAC), and its impairment carried a significantly higher risk of mortality independent of LV EF. They also divided patients into four groups with maintained or reduced LV EF and/or RV EF. The four groups had significantly different survival: both all-cause mortality and cardiac death in patients with reduced RV EF and normal LV EF were significantly higher than in those with reduced LV EF and normal RV EF and did not differ significantly from patients with reduced EF of both ventricles (7). In a secondary analysis of the Atherosclerosis Risk in Communities (ARIC) 3DE substudy, lower RV EF was independently associated with incident heart failure or death in patients free of heart failure at baseline (16). Our group has recently performed a meta-analysis in which we sought to compare the prognostic power of RV EF to conventional echocardiographic parameters (TAPSE, FWLS, FAC). We found that 1 SD reduction in RV EF was robustly associated with adverse cardiopulmonary outcomes, and importantly, RV EF had a superior predictive value compared to the other three parameters (17). Thus, 3DE-derived RV EF seems to be the best parameter among the “standard” echocardiographic metrics of RV function. Nonetheless, similarly to the left ventricle, the assessment of the RV deformation may exceed the added diagnostic and prognostic value of even RV EF. Small cohort studies with various RV pathologies, such as pulmonary hypertension, atrial septal defect, or patients following cardiac surgery, unveiled distinct changes in RV mechanics; still, they were using simple 2D-derived measures of RV function (18–20).

The ReVISION method allows the calculation of the relative contributions of longitudinal, radial, and anteroposterior motion components to global RV function and also 3D longitudinal and circumferential strain, using 3DE-derived models (9). By investigating 300 healthy volunteers using 3DE, Lakatos et al. showed that circumferential EF indexed to global RV EF was clearly dominant compared with longitudinal EF. In contrast to the traditional viewpoint, they found that the relative contribution of the radial and anteroposterior motion directions may be of comparable significance with that of longitudinal shortening in determining global RV function, and standard parameters referring only to longitudinal shortening of the right ventricle may be inadequate to characterize RV function thoroughly (4).

Using the same approach, two studies have already shown the added prognostic value of the RV non-longitudinal functional measures. Kitano et al. examined a relatively large cohort of patients with diverse cardiac diseases, demonstrating that RV 3D strains were associated with the occurrence of hard endpoints even by adjusting for multiple clinical variables (1). Surkova and colleagues enrolled consecutive patients with left-sided heart disease. Even in patients with maintained LV EF, the anteroposterior component of total EF was a significant and independent predictor of outcome (21). Our results add another layer to the evidence about the importance of RV non-longitudinal shortening from a diagnostic and a prognostic point of view. As a previously neglected marker of ventricular systolic performance, circumferential deformation may be able to describe a novel aspect of RV systolic function with established prognostic value. RV GCS may represent a robust, universal biomarker of the status of the entire cardiopulmonary system as it overpowers conventional echocardiographic metrics and is associated with adverse clinical outcomes not just in “classical” right-heart diseases, but also in primary left-heart diseases. The exact pathophysiological processes (ventricular interdependence, RV pressure overload, etc.) that result in the deterioration of circumferential shortening and the potential usefulness of RV GCS for screening purposes remain to be investigated.

### Limitations

Our study has several limitations that have to be acknowledged. First, an inherent limitation is its retrospective, single-center nature. The mixed cohort of patients with some etiologies of left-sided cardiac diseases might not represent the actual patient population treated at a tertiary clinic. However, patients with HFrEF and valvular heart diseases were dominantly included, who are of crucial clinical importance. HTX recipients may represent a unique population in this regard, therefore, we have performed further calculations (see Supplementary Results) to confirm that they do not bias our results. Second, 3DE-based quantifications are not the gold standard; nevertheless, the software packages are well-validated clinically and against cardiac MRI. Calculating RV circumferential strain is quite a novel approach; therefore, literature data are scarce. Third, due to the lack of cause-specific mortality data, we could not investigate the association between the 3DE-derived parameters and cardiac death. The event number has limited the number of covariates in the multivariate models. Lastly, the validity of our results should be tested in prospective outcome studies in different clinical scenarios.

### Conclusions

Biventricular circumferential shortening holds important prognostic value to adverse clinical outcomes. RV GCS is a powerful and independent predictor of all-cause mortality in patients with left-sided cardiac disease. Our study highlights the clinical value of 3DE-derived parameters of myocardial mechanics.

## Data availability statement

The raw data supporting the conclusions of this article will be made available by the authors, without undue reservation.

## Ethics statement

The studies involving human participants were reviewed and the study was approved by the Semmelweis University Regional and Institutional Committee of Science and Research Ethics (approval no. 190/2020). Written informed consent for participation was not required for this study in accordance with the national legislation and the institutional requirements.

## Author contributions

MTol, AFá, and AKov performed measurements, interpreted results, and drafted the manuscript. MTok, ZL, KS, and AFe performed measurements. MTok, BL, AA, ZL, KS, AFe, WS, BV, AKos, ÁS, BS, and BM reviewed and approved the manuscript. BL, AA, WS, BV, AKos, BS, and AKov enrolled, followed-up, and investigated patients. AKov and BM secured grant support. AFá, MTok, and ÁS performed statistical analysis. AFá prepared figures. All authors contributed to the article and approved the submitted version.
