# Supplementary material for: There is more than just longitudinal strain: Prognostic significance of biventricular circumferential mechanics
Source: Front Cardiovasc Med. 2023 Feb 16;10:1082725. doi: 10.3389/fcvm.2023.1082725 (PMC9978526; doi:10.3389/fcvm.2023.1082725)
Supplement: Supplementary file 1 [file Data_Sheet_1.docx]

**There Is More Than Just Longitudinal Strain:
Prognostic Significance of Biventricular Circumferential Mechanics**

SUPPLEMENTARY MATERIAL

Supplementary Table 1. 2D echocardiographic parameters

|  | **Overall**  **(n=357)** | **Alive**  **(n=302)** | **Dead**  **(n=55)** | **p** |
| --- | --- | --- | --- | --- |
| **LVIDd (mm)** | 53.8±9.8 | 53.5±9.7 | 55.5±10.0 | 0.157 |
| **LVIDs (mm)** | 42.0±14.0 | 41.2±13.8 | 45.8±14.3 | 0.073 |
| **IVSd (mm)** | 11.5±2.6 | 11.6±2.6 | 11.1±2.6 | 0.185 |
| **PWd (mm)** | 10.2±2.2 | 10.2±2.1 | 10.1±2.8 | 0.556 |
| **RWT (%)** | 0.40±0.14 | 0.40±0.13 | 0.39±0.18 | 0.553 |
| **LV Mi (g/m^2^)** | 120.3±35.8 | 119.9±36.5 | 122.5±32.1 | 0.632 |
| **E (cm/s)** | 98.4±34.2 | 97.3±32.3 | 104.4±43.1 | 0.179 |
| **A (cm/s)** | 72.0±30.8 | 73.2±30.5 | 64.1±31.5 | 0.095 |
| **E/A** | 1.52±0.71 | 1.50±0.70 | 1.71±0.79 | 0.097 |
| **DT (ms)** | 176.6±58.6 | 177.9±58.9 | 169.1±56.7 | 0.379 |
| **Mitral lateral s′ (cm/s)** | 8.4±3.0 | 8.6±2.9 | 6.9±2.7 | **<0.001** |
| **Mitral lateral e′ (cm/s)** | 10.5±3.9 | 10.6±3.9 | 9.8±3.5 | 0.186 |
| **Mitral lateral a′ (cm/s)** | 7.9±3.2 | 8.2±3.3 | 6.1±2.3 | **<0.001** |
| **Mitral medial s′ (cm/s)** | 6.7±2.3 | 6.8±2.2 | 5.7±2.2 | **0.003** |
| **Mitral medial e′ (cm/s)** | 7.2±2.9 | 7.4±2.9 | 6.1±2.6 | **0.013** |
| **Mitral medial a′ (cm/s)** | 7.3±2.6 | 7.4±2.6 | 6.2±2.6 | **0.020** |
| **E/e′ average** | 12.1±6.4 | 11.8±6.1 | 13.6±7.8 | 0.093 |
| **LAVi (ml/m^2^)** | 46.6±19.5 | 46.4±20.3 | 48.0±15.1 | 0.601 |
| **RVd (mm)** | 35.5±5.9 | 35.2±5.9 | 36.9±6.0 | 0.080 |
| **RVSP (mmHg)** | 40.6±13.8 | 39.6±13.7 | 45.1±13.5 | 0.059 |
| **TAPSE (mm)** | 19.3±6.3 | 19.7±6.4 | 17.3±5.2 | **0.011** |
| **FAC (%)** | 42.9±9.2 | 43.3±9.0 | 40.9±9.7 | 0.079 |
| **RVSLS (%)** | -14.3±6.1 | -14.6±6.0 | -12.2±6.5 | **0.008** |
| **RVFWLS (%)** | -24.0±6.6 | -24.5±6.6 | -21.4±6.2 | **0.002** |
| **RAVi (ml/m^2^)** | 33.6±15.7 | 32.6±14.9 | 38.8±18.5 | **0.011** |

Continuous variables are presented as means ± SD, categorical variables are reported as frequencies (%). A: mitral inflow velocity during atrial contraction, a’: peak late (atrial) diastolic annular velocity, DT: deceleration time, E: early diastolic mitral inflow velocity, e’: early diastolic annular velocity, FAC: fractional area change, IVSd: interventricular septal thickness at end-diastole, LAVi: left atrial volume index, LV: left ventricle, LVIDd: LV internal diameter at end-diastole, LVIDs: LV internal diameter at end-systole, Mi: mass index, PWd: posterior wall thickness at end-diastole, RAVi: right atrial volume index, RV: right ventricle, RVd: RV basal diameter, RVFWLS: RV free wall longitudinal strain, RVSLS: RV septal longitudinal strain, RVSP: RV systolic pressure, RWT: relative wall thickness, s’: systolic annular velocity, TAPSE: tricuspid annular plane systolic excursion

Supplementary Table 2. Factors associated with all-cause mortality using univariable Cox regression

| **Univariable Cox regression** | | |
| --- | --- | --- |
|  | **HR [95% CI]** | **p** |
| **Clinical characteristics** | | |
| **Age** | 1.049 [1.024 - 1.074] | **<0.001** |
| **Sex (male)** | 0.840 [0.473 - 1.490] | 0.550 |
| **Height** | 0.981 [0.952 - 1.010] | 0.195 |
| **Weight** | 0.995 [0.977 - 1.013] | 0.595 |
| **BSA** | 0.617 [0.866 - 2.787] | 0.450 |
| **BMI** | 0.844 [0.739 - 0.964] | **0.012** |
| **Systolic blood pressure** | 1.007 [0.988 - 1.026] | 0.494 |
| **Diastolic blood pressure** | 1.007 [0.983 - 1.030] | 0.584 |
| **Heart rate** | 0.988 [0.959 - 1.018] | 0.439 |
| **History of smoking** | 1.553 [0.176 - 2.160] | 0.140 |
| **COPD** | 1.289 [0.583 - 2.852] | 0.530 |
| **Diabetes** | 1.662 [0.963 - 2.868] | 0.068 |
| **History of atrial fibrillation** | 2.654 [1.548 - 4.550] | **<0.001** |
| **Hypertension** | 1.350 [0.723 - 2.519] | 0.346 |
| **Coronary artery disease** | 3.051 [1.771 - 5.256] | **<0.001** |
| **GFR** | 0.985 [0.971 - 0.999] | **0.038** |
| **Creatinine** | 1.006 [1.001 - 1.011] | **0.030** |
| **Hgb** | 0.949 [0.837 - 1.077] | 0.419 |
| **CRP** | 1.014 [0.996 - 1.031] | 0.121 |
| **2D echocardiographic parameters** | | |
| **LVIDd** | 1.021 [0.995 - 1.048] | 0.117 |
| **LVIDs** | 1.015 [0.992 - 1.038] | 0.210 |
| **IVSd** | 0.927 [0.828 - 1.037] | 0.187 |
| **PWd** | 0.985 [0.860 - 1.127] | 0.822 |
| **RWT** | 0.578 [0.062 - 5.382] | 0.630 |
| **LV Mi** | 1.003 [0.995 - 1.010] | 0.452 |
| **E** | 1.008 [1.000 - 1.016] | 0.053 |
| **A** | 0.996 [0.983 - 1.008] | 0.497 |
| **E/A** | 1.303 [0.814 - 2.086] | 0.270 |
| **DT** | 0.998 [0.992 - 1.003] | 0.442 |
| **Mitral lateral s*’*** | 0.758 [0.672 - 0.854] | **<0.001** |
| **Mitral lateral e*'*** | 0.913 [0.842 - 0.989] | **0.026** |
| **Mitral lateral a*'*** | 0.781 [0.678 - 0.900] | **0.001** |
| **Mitral medial s*'*** | 0.731 [0.621 - 0.861] | **<0.001** |
| **Mitral medial e'** | 0.804 [0.697 - 0.928] | **0.003** |
| **Mitral medial a*'*** | 0.839 [0.712 - 0.989] | **0.036** |
| **E/e*’* average** | 1.062 [1.021 - 1.104] | **0.003** |
| **LAVi** | 1.006 [0.993 - 1.020] | 0.379 |
| **RVd** | 1.043 [0.995 - 1.094] | 0.079 |
| **RVSP** | 1.022 [1.001 - 1.044] | **0.043** |
| **TAPSE** | 0.952 [0.910 - 0.997] | **0.038** |
| **FAC** | 0.970 [0.944 - 0.998] | **0.035** |
| **RVSLS** | 1.072 [1.026 - 1.121] | **0.002** |
| **RVFWLS** | 1.070 [1.027 - 1.115] | **0.001** |
| **RAVi** | 1.021 [1.006 - 1.037] | **0.006** |
| **3D echocardiographic parameters** | | |
| **LV EDVi** | 1.009 [1.002 - 1.017] | **0.016** |
| **LV ESVi** | 1.012 [1.005 - 1.020] | **0.001** |
| **LV SVi** | 0.984 [0.963 - 1.005] | 0.143 |
| **LV Mi** | 1.010 [1.003 - 1.017] | **0.005** |
| **LV EF** | 0.968 [0.953 - 0.983] | **<0.001** |
| **LV GLS** | 1.102 [1.054 - 1.153] | **<0.001** |
| **LV GCS** | 1.056 [1.027 - 1.085] | **<0.001** |
| **RV EDVi** | 1.011 [1.001 - 1.021] | **0.038** |
| **RV ESVi** | 1.018 [1.006 - 1.029] | **0.003** |
| **RV SVi** | 0.987 [0.957 - 1.019] | 0.431 |
| **RV EF** | 0.953 [0.931 - 0.976] | **<0.001** |
| **RV GLS** | 1.116 [1.060 - 1.174] | **<0.001** |
| **RV GCS** | 1.115 [1.068 - 1.164] | **<0.001** |

A: mitral inflow velocity during atrial contraction, a’: peak late (atrial) diastolic annular velocity, BMI: body mass index, BSA: body surface area, CI: confidence interval, COPD: chronic obstructive pulmonary disease, CRP: C-reactive protein, DT: deceleration time, E: early diastolic mitral inflow velocity, e’: early diastolic annular velocity, EDVi: end-diastolic volume index, EF: ejection fraction, ESVi: end-systolic volume index, FAC: fractional area change, GCS: global circumferential strain, GFR: glomerular filtration rate, GLS: global longitudinal strain, Hgb: hemoglobin, HR: hazard ratio, IVSd: interventricular septal thickness at end-diastole, LAVi: left atrial volume index, LV: left ventricle, LVIDd: LV internal diameter at end-diastole, LVIDs: LV internal diameter at end-systole, Mi: mass index, PWd: posterior wall thickness at end-diastole, RAVi: right atrial volume index, RV: right ventricle, RVd: RV basal diameter, RVFWLS: RV free wall longitudinal strain, RVSLS: RV septal longitudinal strain, RVSP: RV systolic pressure, RWT: relative wall thickness, s’: systolic annular velocity, SVi: stroke volume index, TAPSE: tricuspid annular plane systolic excursion

Supplementary Table 3. Factors associated with all-cause mortality using step-by-step multivariate Cox regression analysis.

|  | **STEP I** | | **STEP II^*^** | | | | | | **STEP III^**^** | | | | | |
| --- | --- | --- | --- | --- | --- | --- | --- | --- | --- | --- | --- | --- | --- | --- |
|  | **Model 0** | | **Model 1** | | **Model 2** | | **Model 3** | | **Model 4** | | **Model 5** | | **Model 6** | |
|  | **HR [95% CI]** | **p** | **HR [95% CI]** | **p** | **HR [95% CI]** | **p** | **HR [95% CI]** | **p** | **HR [95% CI]** | **p** | **HR [95% CI]** | **p** | **HR [95% CI]** | **p** |
| **Age** | 1.045 [1.021 - 1.070] | **<0.001** | 1.035 [1.010 - 1.061] | **0.006** | 1.035 [1.011 - 1.060] | **0.005** | 1.035  [1.010 - 1.061] | **0.006** | 1.037 [1.012 - 1.062] | **0.004** | 1.039 [1.014 - 1.064] | **0.002** | 1.036 [1.012 - 1.061] | **0.004** |
| **Sex** | 0.866 [0.476 - 1.576] | 0.637 | 0.738 [0.401 - 1.359] | 0.329 | 0.771 [0.421 - 1.411] | 0.400 | 0.741 [0.401 - 1.370] | 0.339 | 0.732 [0.398 - 1.345] | 0.315 | 0.670 [0.362 - 1.242] | 0.203 | 0.696 [0.379 - 1.279] | 0.243 |
| **Creatinine^#^** | 1.006 [1.000 - 1.012] | **0.047** | 1.005 [0.999 - 1.011] | 0.097 | 1.004 [0.998 - 1.011] | 0.152 | 1.005 [0.999 - 1.011] | 0.083 | 1.005 [0.999 - 1.011] | 0.141 | 1.004 [0.998 - 1.010] | 0.200 | 1.005 [0.999 - 1.012] | 0.090 |
| **LV EF** | - | | 0.978 [0.961 - 0.995] | **0.011** | - | | - | | - | | - | | - | |
| **LV GLS** | - | | - | | 1.071 [1.021 - 1.122] | **0.005** | - | | 1.038 [0.976 - 1.104] | 0.236 | 1.014 [0.953 - 1.079] | 0.662 | 1.016 [0.962 - 1.074] | 0.567 |
| **LV GCS** | - | | - | | - | | 1.037 [1.006 - 1.069] | **0.020** | - | | - | | - | |
| **RV EF** | - | | - | | - | | - | | 0.975  [0.942 - 1.009] | 0.141 | - | | - | |
| **RV GLS** | - | | - | | - | | - | | - | | 1.098 [1.020 - 1.183] | **0.013** | - | |
| **RV GCS** | - | | - | | - | | - | | - | | - | | 1.090 [1.032 - 1.152] | **0.002** |
|  |  |  |  |  |  |  |  |  |  |  |  |  |  |  |
| **AIC** | 566.7 | | 562.4 | | **560.5** | | 563.3 | | 560.4 | | 556.6 | | **553.3** | |

* In Step, II LV functional parameters were added one by one due to their collinearity. ** In Step III, RV functional parameters were added one by one due to their collinearity. ^#^creatinine levels were available in 341 patients

AIC: Akaike information criterion, CI: confidence interval, EF: ejection fraction, GCS: global circumferential strain, GLS: global longitudinal strain, HR: hazard ratio, LV: left ventricle, RV: right ventricle

Supplementary Table 4. Akaike information criterion (AIC) values of multivariate Cox regression models

| **Step I** | **Step II** | **Step III** | **AIC** |
| --- | --- | --- | --- |
| **Age + sex + creatinine** | +LV EF | +RV EF | 561.4 |
|  |  | +RV GLS | 556.8 |
|  |  | +RV GCS | 553.6 |
|  | ***+LV GLS*** | +RV EF | 560.4 |
|  |  | +RV GLS | 556.6 |
|  |  | ***+RV GCS*** | ***553.3*** |
|  | +LV GCS | +RV EF | 561.5 |
|  |  | +RV GLS | 556.8 |
|  |  | +RV GCS | 553.6 |

EF: ejection fraction, GCS: global circumferential strain, GLS: global longitudinal strain, LV: left ventricle, RV: right ventricle

Supplementary Table 5. Independent predictors of all-cause mortality identified using multivariable Cox regression

| **Multivariable Cox regression** | | |
| --- | --- | --- |
|  | **HR [95% CI]** | **p** |
| **Age** | 1.034 [1.009-1.060] | **0.007** |
| **Creatinine^#^** | 1.004 [0.998-1.010] | 0.209 |
| **Coronary artery disease** | 1.436 [0.760-2.712] | 0.265 |
| **LV GLS** | 1.007 [0.950-1.068] | 0.805 |
| **RV GCS** | 1.085 [1.029-1.145] | **0.003** |

^#^creatinine levels were available in 341 patients
CI: confidence interval, HR: hazard ratio, LV GLS: left ventricular global longitudinal strain, RV GCS: right ventricular global circumferential strain

Supplementary Table 6. Independent predictors of all-cause mortality identified using multivariable Cox regression

| **Multivariable Cox regression** | | |
| --- | --- | --- |
|  | **HR [95% CI]** | **p** |
| **Age** | 1.030 [1.005-1.056] | **0.021** |
| **Creatinine^#^** | 1.004 [0.998-1.010] | 0.151 |
| **History of atrial fibrillation** | 1.943 [1.097-3.443] | **0.023** |
| **LV GLS** | 1.014 [0.960-1.071] | 0.620 |
| **RV GCS** | 1.082 [1.025-1.141] | **0.004** |

^#^creatinine levels were available in 341 patients
CI: confidence interval, HR: hazard ratio, LV GLS: left ventricular global longitudinal strain, RV GCS: right ventricular global circumferential strain

Supplementary Table 7. Receiver-operating characteristic analysis of 2D and 3D echocardiographic parameters to discriminate patients with or without adverse outcomes

|  | **AUC [95% CI]** | **p** |
| --- | --- | --- |
| **LVIDd** | 0.576 [0.492 - 0.660] | 0.073 |
| **LVIDs** | 0.605 [0.494 - 0.716] | 0.050 |
| **IVSd** | 0.561 [0.475 - 0.646] | 0.152 |
| **PWd** | 0.561 [0.472 - 0.651] | 0.148 |
| **RWT** | 0.582 [0.490 - 0.674] | 0.053 |
| **LV Mi** | 0.525 [0.440 - 0.611] | 0.570 |
| **E** | 0.537 [0.440 - 0.634] | 0.411 |
| **DT** | 0.569 [0.480 - 0.657] | 0.129 |
| **E/e*’* average** | 0.559 [0.464 - 0.654] | 0.210 |
| **LAVi** | 0.554 [0.475 - 0.633] | 0.234 |
| **RVd** | 0.586 [0.494 - 0.679] | 0.063 |
| **TAPSE** | 0.612 [0.538 - 0.686] | **0.009** |
| **FAC** | 0.584 [0.496 - 0.672] | 0.051 |
| **RVSLS** | 0.609 [0.526 - 0.692] | **0.011** |
| **RVFWLS** | 0.632 [0.552 - 0.711] | **0.002** |
| **RAVi** | 0.606 [0.519 - 0.693] | **0.019** |
| **LV EDVi** | 0.632 [0.549 - 0.714] | **0.003** |
| **LV ESVi** | 0.663 [0.579 - 0.747] | **<0.001** |
| **LV SVi** | 0.521 [0.435 - 0.608] | 0.632 |
| **LV Mi** | 0.591 [0.506 - 0.677] | **0.043** |
| **LV EF** | 0.629 [0.549 - 0.708] | **0.002** |
| **LV GLS** | 0.644 [0.561 - 0.726] | **<0.001** |
| **LV GCS** | 0.621 [0.541 - 0.702] | **0.004** |
| **RV EDVi** | 0.607 [0.522 - 0.692] | **0.017** |
| **RV ESVi** | 0.651 [0.569 - 0.733] | **<0.001** |
| **RV SVi** | 0.507 [0.426 - 0.588] | 0.873 |
| **RV EF** | 0.664 [0.586 - 0.741] | **<0.001** |
| **RV GLS** | 0.672 [0.596 - 0.747] | **<0.001** |
| **RV GCS** | 0.690 [0.614 - 0.765] | **<0.001** |

CI: confidence interval, DT: deceleration time, E: early diastolic mitral inflow velocity, e’: early diastolic annular velocity, EDVi: end-diastolic volume index, EF: ejection fraction, ESVi: end-systolic volume index, FAC: fractional area change, GCS: global circumferential strain, GLS: global longitudinal strain, HR: hazard ratio, IVSd: interventricular septal thickness at end-diastole, LAVi: left atrial volume index, LV: left ventricle, LVIDd: LV internal diameter at end-diastole, LVIDs: LV internal diameter at end-systole, Mi: mass index, PWd: posterior wall thickness at end-diastole, RAVi: right atrial volume index, RV: right ventricle, RVd: RV basal diameter, RVFWLS: RV free wall longitudinal strain, RVSLS: RV septal longitudinal strain, RWT: relative wall thickness, SVi: stroke volume index, TAPSE: tricuspid annular plane systolic excursion

Supplementary Table 8. Hazard ratios of all-cause mortality in the different subgroups

| **Cox proportional-hazards models** | | |
| --- | --- | --- |
|  | **HR [95% CI]** | **p** |
| Group 2 vs. Group 1 | 1.489 [0.453-4.886] | 0.512 |
| Group 3 vs. Group 1 | 3.099 [1.284 - 7.484] | **0.012** |
| Group 4 vs. Group 1 | 5.089 [2.399 -10.793] | **<0.001** |
| Group 3 vs. Group 2 | 2.351 [0.741 -7.459] | 0.147 |
| Group 4 vs. Group 2 | 3.565 [1.256 -10.122] | **0.017** |
| Group 4 vs. Group 3 | 1.515 [0.756 -3.036] | 0.241 |

CI: confidence interval, HR: hazard ratio

Supplementary Table 9. Demographic and clinical characteristics

|  | **Group 1**  **(n=125)** | **Group 2**  **(n=53)** | **Group 3**  **(n=54)** | **Group 4**  **(n=125)** | **Overall p** |
| --- | --- | --- | --- | --- | --- |
| **Baseline demographic characteristics** | | | | | |
| **Age (years)** | 60.4±14.0^bd^ | 67.7±13.4^ac^ | 60.0±15.9^bd^ | 68.4±13.3^ac^ | **<0.001** |
| **Male, n (%)** | 85 (68.0) | 37 (69.8) | 31 (57.4) | 96 (76.8) | 0.071 |
| **BSA (m^2^)** | 1.91±0.22 | 1.96±0.21 | 1.89±0.21 | 1.97±0.23 | 0.113 |
| **BMI (kg/m^2^)** | 26.1±3.5 | 26.6±4.7 | 28.6±3.6 | 27.1±4.8 | 0.263 |
| **Systolic blood pressure (mmHg)** | 130.1±16.8^d^ | 130.9±15.9^d^ | 129.1±21.3 | 122.3±19.0^ab^ | **0.023** |
| **Diastolic blood pressure (mmHg)** | 74.5±16.8 | 74.2±15.7 | 78.3±17.0 | 74.3±14.2 | 0.815 |
| **Heart rate (bpm)** | 77.7±15.1 | 76.1±15.5 | 89.3±9.3 | 76.6±14.2 | 0.093 |
| **Risk factors and medical history** | | | | | |
| **Hypertension, n (%)** | 89 (71.2) | 43 (81.1) | 36 (66.7) | 92 (73.6) | 0.378 |
| **History of smoking, n (%)** | 26 (20.8) | 13 (24.5) | 6 (11.1) | 37 (29.6) | 0.050 |
| **COPD, n (%)** | 13 (10.4) | 5 (9.4) | 7 (13.0) | 15 (12.0) | 0.914 |
| **Diabetes, n (%)** | 27 (21.6)^d^ | 19 (35.8)^c^ | 10 (18.5)^bd^ | 43 (34.4)^ac^ | **0.027** |
| **History of atrial fibrillation, n (%)** | 34 (27.2)^d^ | 19 (35.8) | 12 (22.2)^d^ | 51 (40.8)^ab^ | **0.033** |
| **PM, n (%)** | 2 (1.6)^bd^ | 10 (18.9)^ac^ | 2 (3.7)^bd^ | 35 (28.0)^ac^ | **<0.001** |
| **ICD, n (%)** | 1 (0.8)^d^ | 4 (7.5)^d^ | 0 (0.0)^d^ | 28 (22.4)^abc^ | **<0.001** |
| **CRT, n (%)** | 0 (0.0)^d^ | 3 (5.7) | 0 (0.0)^d^ | 12 (9.6)^ac^ | **0.001** |
| **CAD, n (%)** | 10 (8.0)^bd^ | 17 (32.1)^ac^ | 1 (1.9)^bd^ | 49 (39.2)^ac^ | **<0.001** |
| - **Previous CABG, n (%)** | 0 (0.0)^d^ | 2 (3.8)^d^ | 0 (0.0)^d^ | 17 (13.6)^abc^ | **<0.001** |
| - **Previous PCI, n (%)** | 9 (7.2)^bd^ | 14 (26.4)^ac^ | 1 (1.9)^bd^ | 43 (34.4)^ac^ | **<0.001** |
| - **Previous AMI, n (%)** | 1 (0.8)^bd^ | 10 (18.9)^acd^ | 0 (0.0)^bd^ | 37 (29.6)^abc^ | **<0.001** |
| **Laboratory parameters** | | | | | |
| **GFR (mL/min/1.73m^2^)** | 64.6±21.5 | 59.5±20.3 | 63.6±19.5 | 57.8±16.7 | 0.083 |
| **Creatinine (μmol/L)** | 94.8±36.3 | 103.0±51.6 | 97.3±40.9 | 108.4±42.2 | 0.075 |
| **Hgb (g/dL)** | 13.1±2.2^c^ | 13.0±1.7^c^ | 12.0±2.1^abd^ | 13.0±2.1^c^ | **0.010** |
| **CRP (mg/L)** | 4.3±9.0 | 8.4±16.9 | 7.2±10.7 | 8.2±12.2 | 0.060 |

Continuous variables are presented as means ± SD, categorical variables are reported as frequencies (%). a: p < 0.05 vs. Group 1, b: p < 0.05 vs. Group 2, c: p < 0.05 vs. Group 3, d: p < 0.05 vs. Group 4

AMI: acute myocardial infarction, BMI: body mass index, BSA: body surface area, CABG: coronary artery bypass grafting, CAD: coronary artery disease, COPD: chronic obstructive pulmonary disease, CRP: C-reactive protein, CRT: cardiac resynchronization therapy, GFR: glomerular filtration rate, Hgb: hemoglobin, ICD: implantable cardioverter defibrillator, PCI: percutaneous coronary intervention, PM: pacemaker

Supplementary Table 10. 2D echocardiographic parameters

|  | **Group 1**  **(n=125)** | **Group 2**  **(n=53)** | **Group 3**  **(n=54)** | **Group 4**  **(n=125)** | **Overall p** |
| --- | --- | --- | --- | --- | --- |
| **LVIDd (mm)** | 51.8±8.7^d^ | 51.4±7.6^d^ | 49.4±9.1^d^ | 58.7±9.9^abc^ | **<0.001** |
| **LVIDs (mm)** | 31.8±8.6^bd^ | 39.8±10.0^acd^ | 28.8±7.7^bd^ | 49.7±13.2^abc^ | **<0.001** |
| **IVSd (mm)** | 11.4±2.4^b^ | 12.5±3.2^acd^ | 11.4±1.9^b^ | 11.3±2.6^b^ | **0.020** |
| **PWd (mm)** | 10.0±1.9 | 10.5±2.8 | 10.0±1.6 | 10.4±2.5 | 0.349 |
| **RWT (%)** | 0.40±0.13 | 0.42±0.16^d^ | 0.42±0.13^d^ | 0.37±0.14^bc^ | **0.043** |
| **LV Mi (g/m^2^)** | 113.3±33.8^d^ | 119.2±38.3^cd^ | 103.3±30.2^bd^ | 136.2±33.2^abc^ | **<0.001** |
| **E (cm/s)** | 103.4±32.5^d^ | 98.5±35.2 | 103.3±29.5^d^ | 90.8±36.3^ac^ | **0.028** |
| **A (cm/s)** | 68.1±28.2 | 80.5±35.8 | 67.8±27.4 | 75.2±32.4 | 0.102 |
| **E/A** | 1.69±0.63^bd^ | 1.35±0.73^a^ | 1.62±0.58^d^ | 1.36±0.80^ac^ | **0.004** |
| **DT (ms)** | 181.6±51.1 | 183.9±53.5 | 172.3±74.4 | 170.3±59.9 | 0.457 |
| **Mitral lateral s′ (cm/s)** | 9.9±2.4^bd^ | 7.7±2.6^acd^ | 9.8±2.9^bd^ | 6.4±2.3^abc^ | **<0.001** |
| **Mitral lateral e′ (cm/s)** | 12.0±3.5^bd^ | 9.3±3.9^ac^ | 12.5±4.0^bd^ | 8.6±3.1^ac^ | **<0.001** |
| **Mitral lateral a′ (cm/s)** | 8.5±3.0^d^ | 8.2±3.5^d^ | 8.4±3.1^d^ | 6.9±3.1^abc^ | **0.004** |
| **Mitral medial s′ (cm/s)** | 8.2±1.7^bd^ | 6.0±1.7^acd^ | 7.8±2.3^bd^ | 5.1±1.8^abc^ | **<0.001** |
| **Mitral medial e′ (cm/s)** | 8.7±2.7^bd^ | 6.5±3.3^ac^ | 8.1±2.6^bd^ | 5.7±1.9^ac^ | **<0.001** |
| **Mitral medial a′ (cm/s)** | 7.9±2.2^d^ | 7.2±2.8 | 7.8±2.8^d^ | 6.3±2.7^ac^ | **<0.001** |
| **E/e′ average** | 10.5±4.7^bd^ | 13.7±7.9^ac^ | 10.7±5.1^bd^ | 13.6±7.2^ac^ | **<0.001** |
| **LAVi (ml/m^2^)** | 48.1±21.4 | 46.4±20.2 | 43.3±17.5 | 46.8±18.3 | 0.548 |
| **RVd (mm)** | 34.0±4.5^d^ | 34.8±5.9^d^ | 33.2±4.5^d^ | 38.5±6.8^abc^ | **<0.001** |
| **RVSP (mmHg)** | 39.3±13.7 | 36.5±9.9 | 42.2±17.1 | 43.6±12.7 | 0.230 |
| **TAPSE (mm)** | 22.4±6.6^bcd^ | 19.5±5.6^ad^ | 17.6±6.2^a^ | 16.9±4.7^ab^ | **<0.001** |
| **FAC (%)** | 46.0±6.9^d^ | 47.2±7.7^cd^ | 43.8±8.7^bd^ | 37.7±9.5^abc^ | **<0.001** |
| **RVSLS (%)** | -17.4±5.2^bd^ | -13.5±4.3^acd^ | -16.7±6.2^bd^ | -10.4±5.3^abc^ | **<0.001** |
| **RVFWLS (%)** | -27.4±5.1^cd^ | -26.0±6.0^cd^ | -23.7±5.9^abd^ | -19.9±6.3^abc^ | **<0.001** |
| **RAVi (ml/m^2^)** | 30.1±12.6^d^ | 32.2±17.6^d^ | 31.4±14.0^d^ | 38.8±17.3^abc^ | **<0.001** |

Continuous variables are presented as means ± SD, categorical variables are reported as frequencies (%). a: p < 0.05 vs. Group 1, b: p < 0.05 vs. Group 2, c: p < 0.05 vs. Group 3, d: p < 0.05 vs. Group 4.

A: mitral inflow velocity during atrial contraction, a’: peak late (atrial) diastolic annular velocity, DT: deceleration time, E: early diastolic mitral inflow velocity, e’: early diastolic annular velocity, FAC: fractional area change, IVSd: interventricular septal thickness at end-diastole, LAVi: left atrial volume index, LV: left ventricle, LVIDd: LV internal diameter at end-diastole, LVIDs: LV internal diameter at end-systole, Mi: mass index, PWd: posterior wall thickness at end-diastole, RAVi: right atrial volume index, RV: right ventricle, RVd: RV basal diameter, RVFWLS: RV free wall longitudinal strain, RVSLS: RV septal longitudinal strain, RVSP: RV systolic pressure, RWT: relative wall thickness, s’: systolic annular velocity, TAPSE: tricuspid annular plane systolic excursion

Supplementary Table 11. 3D echocardiographic parameters

|  | **Group 1**  **(n=125)** | **Group 2**  **(n=53)** | **Group 3**  **(n=54)** | **Group 4**  **(n=125)** | **Overall p** |
| --- | --- | --- | --- | --- | --- |
| **Left ventricle** | | | | | |
| **LV EDVi (ml/m^2^)** | 74.7±24.9^d^ | 74.1±26.8^d^ | 66.9±21.4^d^ | 100.6±36.8^abc^ | **<0.001** |
| **LV ESVi (ml/m^2^)** | 28.4±9.2^bd^ | 43.9±24.3^acd^ | 26.5±9.0^bd^ | 70.0±35.5^abc^ | **<0.001** |
| **LV SVi (ml/m^2^)** | 46.3±16.8^bcd^ | 30.3±8.1^ac^ | 40.4±13.5^abd^ | 30.6±8.2^ac^ | **<0.001** |
| **LV Mi (g/m^2^)** | 84.4±25.3^bd^ | 109.2±39.0^acd^ | 82.5±24.1^bd^ | 127.9±34.8^abc^ | **<0.001** |
| **LV EF (%)** | 60.8±4.9^bd^ | 45.2±12.4^acd^ | 60.3±5.4^bd^ | 34.0±13.4^abc^ | **<0.001** |
| **LV GLS (%)** | -20.5±3.2^bd^ | -12.2±3.1^acd^ | -19.4±3.4^bd^ | -9.5±3.5^abc^ | **<0.001** |
| **LV GCS (%)** | -30.3±3.8^bd^ | -21.9±7.6^acd^ | -30.6±4.1^bd^ | -15.6±7.9^abc^ | **<0.001** |
| **Right ventricle** | | | | | |
| **RV EDVi (ml/m^2^)** | 65.2±16.8^d^ | 61.6±19.5^d^ | 65.0±17.8^d^ | 81.1±28.9^abc^ | **<0.001** |
| **RV ESVi (ml/m^2^)** | 29.6±8.5^d^ | 30.3±12.4^d^ | 33.4±11.1^d^ | 50.2±23.8^abc^ | **<0.001** |
| **RV SVi (ml/m^2^)** | 35.6±9.4^bcd^ | 31.3±8.5^a^ | 31.6±8.1^a^ | 30.9±8.6^a^ | **0.001** |
| **RV EF (%)** | 54.7±4.4^bcd^ | 52.0±5.9^acd^ | 48.8±5.9^abd^ | 40.1±9.5^abc^ | **<0.001** |
| **RV GLS (%)** | -20.3±3.9^bcd^ | -16.2±3.2^ad^ | -17.2±4.0^ad^ | -12.3±3.9^abc^ | **<0.001** |
| **RV GCS (%)** | -23.2±3.5^bcd^ | -21.4±3.2^acd^ | -14.2±3.6^abd^ | -12.1±3.6^abc^ | **<0.001** |

Continuous variables are presented as means ± SD, categorical variables are reported as frequencies (%). a: p < 0.05 vs. Group 1, b: p < 0.05 vs. Group 2, c: p < 0.05 vs. Group 3, d: p < 0.05 vs. Group 4

EDVi: end-diastolic volume index, EF: ejection fraction, ESVi: end-systolic volume index, GCS: global circumferential strain, GLS: global longitudinal strain, LV: left ventricle, Mi: mass index, RV: right ventricle, SVi: stroke volume index

Supplementary Table 12. Intra- and interobserver variability of 3D right ventricular functional metrics

|  | **Intraclass correlation coefficient [95% CI]** | |
| --- | --- | --- |
|  | **Intraobserver** | **Interobserver** |
| **RV EF** | 0.966 [0.902 - 0.988] | 0.971 [0.918 - 0.990] |
| **RV GLS** | 0.935 [0.823 - 0.978] | 0.767 [0.437 - 0.915] |
| **RV GCS** | 0.888 [0.700 - 0.961] | 0.753 [0.416 - 0.909] |

CI: confidence interval, EF: ejection fraction, GCS: global circumferential strain, GLS: global longitudinal strain, RV: right ventricle

**Supplementary Results**

We dichotomized our patient population to HTX patients vs. Non-HTX patients.

Using univariable Cox regression analysis on the entire population, HTX was an independent predictor of outcome (HR, 0.431 [95% CI, 0.217-0.858], p<0.05): being an HTX recipient was associated with a lower risk of experiencing adverse outcomes.

Then, we assessed its prognostic value in the established multivariable model including HTX dichotomization instead of sex. Therefore, we included age, HTX, creatinine levels, LV GLS, and RV GCS. Using multivariable Cox regression analysis, age (HR, 1.036 [95% CI, 1.008-1.065], p=0.013) and RV GCS (HR, 1.086 [95% CI, 1.029-1.145], p=0.003) were the only significant and independent predictors of outcome, whereas HTX (HR, 0.925 [95% CI, 0.389-2.202], p= 0.861) was not.

Furthermore, when we tested the different subgroups (Group 1, Group 2, Group 3, Group 4) separately, HTX was not significantly associated with the outcomes in any of the four groups using univariable Cox regression.
